# Peer Preference and Executive Functioning Development: Longitudinal Relations Among Females With and Without ADHD

**DOI:** 10.1007/s10802-025-01333-x

**Published:** 2025-05-28

**Authors:** Patricia A. Porter, Yuchen Zhao, Stephen P. Hinshaw

**Affiliations:** 1 Department of Psychology, University of California, Berkeley, 2121 Berkeley Way, Berkeley, CA 94720-1650 USA; 2 Department of Psychology, University of Washington, 119 Guthrie Hall, Seattle, WA 98195-1525 USA; 3 Department of Psychiatry and Behavioral Science, UCSF Weill Institute for Neurosciences, University of California, San Francisco, USA

**Keywords:** ADHD, Executive functioning, Peers, Inhibition

## Abstract

Peer problems are a pervasive issue for children with ADHD, but less is known about the role of peers in the development of executive functioning (EF). We examined the predictive relation between childhood peer preference (i.e., the extent to which one is liked vs. disliked by peers) and the development of various EF skills (response inhibition, working memory, and global EF) from childhood to early adulthood within a diverse female sample enriched for ADHD. We sampled 140 girls diagnosed with ADHD in childhood and 88 neurotypical comparison girls, matched for age and race. Girls were 6–12 years old at baseline and followed for three additional waves across 16 years. Peer preference was assessed via sociometric interviews in childhood; EF data were collected at all waves via neuropsychological tests. Through multilevel modeling, we evaluated relations between childhood peer preference and the development of each EF skill from childhood to early adulthood, adjusting for ADHD diagnostic status, verbal IQ, and socioeconomic status. We found that lower peer preference in childhood (a) was associated with poorer global EF across development and (b) predicted significantly less improvement in response inhibition from childhood to adulthood. Childhood ADHD diagnostic status was also related to lower global EF and response inhibition across development, but unlike peer preference, ADHD was not predictive of differences in EF growth. Secondary analyses revealed that peer rejection, not acceptance, drove these core findings. Findings highlight the influence of childhood peer preference on EF development, particularly response inhibition. We discuss intervention implications.

Important social, physical, and neurobiological transitions occur from childhood to early adulthood, leaving many individuals vulnerable to a range of mental health disorders (Kessler et al., 2007; Steinberg, 2005). Such transitions are related, in part, to dramatic changes in brain structure and function essential for the development of executive functioning (EF; Casey et al., 2008). Although different conceptions exist, EF typically refers to an interconnected set of self-regulatory, cognitive processes responsible for purposeful, goal-directed action, including components such as inhibitory control and working memory (Best & Miller, 2010; Huizinga et al., 2006). EF is crucial to adaptive development across multiple domains of functioning (Hughes & Ensor, 2011; Miller & Hinshaw, 2010; Rinsky & Hinshaw, 2011). In particular, numerous studies highlight the link between EF deficits and attention-deficit/hyperactivity disorder (ADHD), a prevalent neurodevelopmental condition characterized by extreme and impairing levels of inattention and/or hyperactivity/impulsivity (Pievsky & McGrath, 2018; Willcutt et al., 2005). Given the importance of EF, research examining malleable factors promoting adaptive EF development is crucial to informing early transdiagnostic interventions.

An emerging body of research highlights the importance of peer relationships in fostering EF development (for review see Hay et al., 2004 and Pahigiannis & Glos, 2020). For example, using a cross-lagged design, Holmes et al. (2016) found that children experiencing higher peer problems in childhood exhibited lower EF skills years later. Socio-constructivist theorists postulate that peer-rejected children have less opportunity to practice skills important to EF development (Rubin et al., 2006; Tudge & Winterhoff, 1993)—under the assumption that play activities in childhood may provide an important context for the development of working memory and inhibition (Thibodeau et al., 2016; White et al., 2021). Whereas these socio-constructivist models focus on the benefits of social interactions with peers, other models emphasize the role of negative peer experiences in limiting EF. For example, Baumeister et al. (1998) posited that peer rejection threatens an individual’s fundamental need for social belonging, depleting limited cognitive resources and thereby impairing EF processes. This theory is bolstered by multiple experimental manipulation studies showing that individuals randomly assigned to experience peer rejection exhibit poorer performance on subsequent EF tasks, at least in the short term (Baumeister et al., 2005; Hawes et al., 2012; King et al., 2018). Yet longitudinal studies examining the extent of such effects on EF across development are scarce (notably Holmes et al., 2016, discussed above; de Wilde et al., 2016; Lecce et al., 2020; see also Burke et al., 2023; Hernández et al., 2017; Stenseng et al., 2015 on self-regulation more generally). Several questions remain, including whether peer influence on EF development (a) generalizes to clinical populations with high EF deficits, such as those with ADHD; (b) extends beyond childhood; or (c) varies across different EF components, as discussed further below.

## Importance for Individuals With ADHD

Research clearly shows that that peer problems are a pervasive issue for children with ADHD (Hoza et al., 2005; Murray-Close et al., 2010), often attributing this finding to symptoms of the disorder that may elicit peer rejection. Still, a major gap in the literature exists pertaining to how peer relationships may in turn influence EF development for such youth. Longitudinal studies using cross-lagged designs suggest that this relation is likely to be bidirectional: Children with higher ADHD symptoms are more likely to experience peer problems, which in turn predict higher subsequent ADHD symptoms, at least within community samples consisting primarily of typically developing children (Ji et al., 2019; Stenseng et al., 2016; Tseng et al., 2014). However, this effect has yet to be explored within clinical populations enriched for ADHD, particularly regarding the development of EF, using neuropsychological measures that are potentially less biased by factors related to social preference than informant reports of symptoms.

## EF Development Beyond Childhood

Individuals experience significant growth in EF throughout childhood and adolescence (Jurado & Rosselli, 2007), with maturational changes continuing into the mid-20s (De Luca et al., 2003; Ferguson et al., 2021). Yet past research regarding the potential impact of peers on EF has centered on development in childhood (e.g., Burke et al., 2023; de Wilde et al., 2016; Hernández et al., 2017; Lecce et al., 2020; Stenseng et al., 2015, 2016). Some studies show that peer relationships are predictive of emotion regulation in adolescence (e.g., see Farley & Kim-Spoon, 2014 for review), but studies of whether these findings extend to EF processing outside of heightened emotional contexts (i.e., cold EF) are scarce and reveal conflicting results. Notably, Holmes et al. (2016) found that peer problems predicted poorer global EF later in childhood but not adolescence, whereas Ji et al. (2019) found that childhood peer rejection was predictive of adolescent attention problems. Longitudinal evaluation of the association between peer processes and EF across longer timespans is particularly needed within ADHD samples, given differences in their EF developmental trajectories relative to typically developing peers. Despite within-participant improvements in EF across development, most children with ADHD continue to exhibit EF deficits relative to neurotypical youth into adulthood (Gordon & Hinshaw, 2020). Still, significant heterogeneity exists in adolescent and adult symptom trajectories for those with ADHD (Shaw & Sudre, 2021). Given that improvements in EF skills are predictive of decreases in ADHD symptoms (Miller et al., 2013), research on EF development among individuals with ADHD into early adulthood, when EF maturation peaks, is a priority.

## Different EF Components

Although multiple studies suggest that peer relationships affect EF, the literature is clouded by a lack of consistency in the operationalization of EF, with most investigations relying on a single measure of global self-regulation based on informant report. However, distinct EF components emerge across development, such as response inhibition and working memory (McAuley & White, 2011; Miyake & Friedman, 2012), which show different trajectories across the lifespan (Best & Miller, 2010). It is therefore crucial to identify how peer relationships may influence the development of distinct EF measures. Limited as it is, current research reveals mixed findings. For example, Lecce et al. (2020) found that peer acceptance and rejection in middle childhood were predictive of working memory and response inhibition months later, using a cross-lagged design. Yet other investigations did not reveal predictive links between peer relationships and working memory (de Wilde et al., 2016).

## Gender Differences

Finally, the study of peer relationships and EF development may be particularly important to *females* with ADHD, considering gender differences in types of play and responses to rejection. Children tend to affiliate with same-gendered peers in childhood and display varying affinities for distinct types of play by gender, which may differentially influence EF development (Rose & Rudolph, 2006). Additionally, girls with ADHD appear to be at higher risk for peer rejection than boys with the disorder: ADHD is less prevalent among girls and hence characteristics associated with the disorder (e.g., impulsivity, difficulty listening) appear to be less socially accepted within female peer groups (Carlson et al., 1997; Diamantopoulou et al., 2005; Kok et al., 2016). Even more, girls may have a heightened sensitivity to peer rejection compared to boys (Rose & Rudolph, 2006.; see also Hawes et al., 2012, who found that girls randomly assigned to rejection—but not boys—exhibited poorer subsequent EF). Thus, girls with ADHD may be particularly vulnerable to the impacts of peer rejection, making relevant research a priority for this high-risk population (Hinshaw et al., 2022; Young et al., 2020).

## Current Study

We therefore examine predictive relations between childhood peer preference (i.e., the extent to which a child is accepted vs. rejected by their peers) and the development of various EF skills (response inhibition, working memory, and global EF) from childhood to early adulthood, in females with and without ADHD, stringently adjusting for baseline ADHD diagnostic status, verbal IQ, and household socioeconomic status (SES). Whereas past studies have predominantly relied on cross-lagged designs, focusing on inter-individual differences over time, we use a multilevel modeling approach to evaluate individual differences in EF growth across development. Regarding EF components, we focus on (a) response inhibition and (b) working memory, given past findings suggesting their connections with peer relationships (Hawes et al., 2012; Lecce et al., 2020), as well as (c) a global EF measure of planning and organizational skills (Sami et al., 2004). As noted, we adjust for childhood ADHD diagnostic status, verbal IQ, and SES to isolate any effect of peer preference on EF development, as these covariates are related to both peer preference and EF (Hoza et al., 2005; Merz et al., 2019; Murray-Close et al., 2010; Ridge, 2011; Troesch et al., 2016; Willcutt et al., 2005). We focus specifically on verbal (rather than full-scale) IQ, given the importance of communication in peer interactions and to avoid statistical overcontrol, considering full-scale IQ calculations include working memory subtests.

To the best of our knowledge, this is the first study to evaluate the prospective relations between childhood peer preference and EF development extending from childhood to early adulthood within a clinical sample enriched for ADHD. We hypothesize that lower peer preference in childhood will be associated with poorer EF skills across development through early adulthood and will also predict less improvement in EF across this timespan. We highlight predictive linkages with response inhibition, working memory, and global EF, but given the dearth of relevant literature, we do not offer specific hypotheses differentiating predictive associations with these distinct EF components.

## Method

### Participants and Procedure

We utilized data collected from a prospective sample of girls diagnosed with ADHD in childhood (*n* = 140), along with a comparison sample without ADHD (*n* = 88), who participated in the Berkeley Girls with ADHD Longitudinal Study (BGALS). ADHD and comparison samples were group-matched on age and race, as described in Hinshaw (2002). The racial makeup of the sample was 53% European American, 27% African American, 11% Latin American, and 9% Asian American, with a wide range of SES backgrounds. Of the girls with ADHD, 34% presented as predominantly inattentive and 66% as combined (i.e., hyperactive/impulsive and inattentive). The BGALS project was approved by the Committee for Protection of Human Subjects (i.e., the Institutional Review Board) at the University of California, Berkeley. Written consent and assent were obtained from both legal guardians and youth participants, respectively, at each wave.

Per Hinshaw (2002), girls aged 6–12 years were recruited from multiple sources including medical, educational, and mental health settings, to participate in research summer camp programs in 1997, 1998, and 1999. We then performed a battery of screening and diagnostic assessment to determine eligibility for the summer camps. Exclusionary criteria included IQ less than 70; overt neurological damage, psychosis, or pervasive developmental disorder; and medical conditions that prevented participation in the camp. Common psychiatric comorbidities (e.g., oppositional defiant disorder [ODD], conduct disorder [CD], learning disorders, etc.) were allowed to promote generalizability of the ADHD sample. Comparison girls with internalizing disorders and/or ODD were included to avoid creating a supernormal comparison sample.

The summer programs featured a series of classroom, art, drama, and outdoor activities. Groups of 25–26 girls (61% with ADHD, 39% comparison), grouped by age, participated together for each day’s events. Individual, confidential peer sociometric nominations were collected from each participant at the end of Weeks 1, 3, and 5. During pre-camp assessments and the summer program, each girl completed a series of neuropsychological tests, including the Conners’ Continuous Performance Test (Conners, 1995), Wechsler Intelligence Scale for Children (WISC-III; Wechsler, 1991) and Rey-Osterrieth Complex Figure Test (ROCF; Osterrieth, 1944). All neuropsychological testing was performed while girls were not taking stimulant medication and assessors were unaware of participant diagnosis. We asked that girls participate in the summer camps without ADHD medication. The majority did so, but for a minority whose parents requested a medication evaluation (*n* = 27), these participants received their usual stimulant medication for two of the four weeks following the initial week.

Participants were then followed longitudinally approximately every five years across three additional evaluation waves. The age ranges at each wave were as follows—Wave 1: 6–12 years, Wave 2: 11–18 years, Wave 3: 17–25 years, and Wave 4: 22–29 years. At each follow-up, participants completed additional neuropsychological testing, including the Conners’ CPT, backward digit span, and complex figure tests. Across waves, sample retention was excellent (92–95%). Per Owens et al. (2017), the retained sample at Wave 4 was higher in SES and IQ than those not retained, but no significant differences in race, age, or ADHD diagnostic status emerged.

### Measures

***Childhood ADHD Diagnosis.*** Initial screenings included parent- and teacher-report on the Swanson, Nolan, and Pelham Rating Scale (4th ed.; SNAP-IV; Swanson, 1992). Eligibility for the ADHD group was then determined by meeting full diagnostic criteria based on parental report on the Diagnostic Interview Schedule for Children (4th ed.; DISC-IV; Shaffer et al., 2000).

***Response Inhibition.*** This was measured via the Conners’ Continuous Performance Test (Conners, 1995), a computerized task with instructions to press a button when any letter except for ‘X’ is shown—and refrain from hitting the button when ‘X’ is shown. Across 14 min, six trial blocks were shown, with interstimulus intervals set at one, two, or four sec within each block. Unlike many other CPTs, Conners’ has a high signal-to-noise ratio, designed to test inhibition, with 80% of all letters being targets (i.e., non-X stimuli) and only 20% non-targets (i.e., ‘X’s). Response inhibition was assessed using the proportion of commission errors– the number of responses to non-targets (i.e., ‘X’s) out of the total number of non-targets presented, which has high test-retest reliability (0.78–0.82; Shaked et al., 2020; Zabel et al., 2009). We focus on commission errors given their strong association with ADHD symptoms (Epstein et al., 2003) and consistency with other measures of inhibitory control (Shaked et al., 2020).

***Working Memory.*** This was assessed using the Backward Digit Span score from the Digit Span Subtest of the Wechsler Intelligence Scale for Children (WISC-III; Wechsler, 1991) at Waves 1–2 and Wechsler Adult Intelligence Scale (WAIS-III; Wechsler, 1997) at Waves 3–4. In this task, participants are read a series of numbers aloud increasing in length and then asked to immediately repeat the numbers in their reverse presentation order, requiring them to leverage working memory skills to temporarily store and manipulate information. Participant scores are based on the longest number sequence recalled correctly, with higher scores reflecting better working memory. We focus on *backward* digit span, given the importance of manipulation of information to the construct of working memory. This task shows moderate test-retest reliability (0.50–0.75) and good correlations with other measures of working memory and attention (Conway et al., 2005; Wechsler, 1991, 1997).

***Global EF.*** This was evaluated based on performance in the copy condition of the Rey-Osterrieth Complex Figure Test (ROCF; Osterrieth, 1944) at Waves 1, 3, and 4. At Wave 2, the analogous Taylor Complex Figure Test (TCFT; Taylor, 1969) was used to avoid practice effects, which shows similar performance in the copy condition (Kuehn, 1992). In this task, participants view a complex figure and are asked to recreate the image on a piece of paper without tracing or the ability to erase their work. This task captures several EF skills, including planning/organization, working memory, and self-regulation, so we consider it a measure of global EF. Performance during the copy condition (i.e., while looking at the reference image) is significantly correlated with other measures of EF and has shown particular sensitivity in capturing EF deficits related to ADHD (Sami et al., 2004; Watanabe et al., 2005). Trained research assistants coded these figures using the error proportion scoring system (i.e., the number of errors divided by the total number of segments drawn), whereby higher scores indicate greater EF deficits. We utilize the error proportion score as it was is designed to capture global EF skills related to planning and organization (as opposed to focusing on visuo-spatial abilities), as reflected in its strong associations with EF impairments among children with ADHD (Sami et al., 2004). As for inter-rater agreement, intraclass correlations between the pairs of the three primary scorers at each wave ranged from 0.91 to 0.94 for the ROCF and 0.77 to 0.94 for the TCFT.

***Socioeconomic Status (SES).*** This comprised the standardized average of parent-reported maternal education (*M* = 4.79, *SD* = 0.95) and family annual income (*M* = 6.43, *SD* = 2.57) at Wave 1. Highest level of maternal education was rated on scale from 1 (less than 8th grade) to 6 (advanced or professional degree), and income on a scale from 1 (< $10,000) to 9 ($75,000+), as in previous studies (e.g., Owens et al., 2017).

***Verbal IQ.*** This estimated score was based on participant performance on the Vocabulary and Similarities subtests of the WISC-III (Wechsler, 1991) at Wave 1.

***Peer Preference.*** Using standard sociometric procedures, private peer nominations from each participant were collected at the end of Weeks 1, 3, and 5 of each summer program (Coie et al., 1982). Reviewing a picture board composed of head-and-shoulders photographs of all classmates, each girl nominated three peers whom she most liked and three whom she most disliked. Peer acceptance was assessed via the proportion of positive nominations each girl received relative to the number of girls in her classroom—and peer rejection as the proportion of negative nominations received, which were moderately correlated (*r* = -.47, *p* <.01). For consistency with past literature and because of the skewed nature of sociometric rejection and acceptance distributions, we focus on a widely used measure of overall peer preference as the primary predictor, operationalized as the difference between peer acceptance and rejection proportion scores (Coie et al., 1982; Mpofu et al., 2006). We also provide supplementary analyses examining peer acceptance and rejection separately as predictors to parse out these effects. The stability of peer preference across weeks was high (*r* =.85, *p* <.01). For analyses, we used final sociometric nominations at the end of the summer camp (Week 5) to reflect peer preference after prolonged interactions.

Given the atypically high prevalence of nominators with ADHD in our sample, past studies from our lab compared sociometric nominations given by girls with ADHD vs. comparison girls, showing similar overall patterns (see Blachman & Hinshaw, 2002, for detail). That is, girls with ADHD were more rejected than comparisons by all nominators but were rated slightly more positively and slightly less negatively by peers with ADHD than by comparison girls. We thus consider these nominations to be reflective of participant general social preference among peers. Yet our higher prevalence of ADHD nominators may potentially slightly overestimate the level of peer preference of the girls with ADHD (see Discussion).

### Data Analytic Plan

We utilized multilevel models to examine predictors of the trajectories of response inhibition, working memory, and global EF from childhood to early adulthood, nested within individual participants. This approach allowed us to model development across age continuously, accounting for wider participant age ranges at later assessment waves. Participant age was centered at nine years of age (the mean age at Wave 1) for ease of intercept interpretability. First, we evaluated fixed and random age effects by comparing a series of unconditional growth models, testing whether the developmental trajectory of each EF measure was best characterized by linear or quadratic patterns of change and whether each coefficient should be treated as random or fixed. Next, we tested conditional models for each outcome (a) including only covariates (i.e., verbal IQ, SES, ADHD diagnosis) and their interactions with age; and (b) adding childhood peer preference and its interaction with age as predictors, where continuous childhood predictors were grand mean centered. Finally, we reconducted these conditional models, evaluating childhood peer rejection and acceptance as separate predictors (instead of peer preference). Goodness of fit was assessed using the log likelihood (LL), Akaike information criterion (AIC), and adjusted chi-square difference tests based on LL values. Missing data were handled using maximum likelihood estimation; all analyses were conducted using the R lme4 package (Bates et al., 2015).

## Results

### Preliminary Analyses

***Missing Data.*** Regarding EF data, 28 participants (12.3%) were missing CPT and ROCF data at Wave 4 and 20 (8.8%) were missing backward digit span data. To evaluate the representativeness of the retained sample, we compared those with vs. without Wave 4 EF data across key variables and demographics at Wave 1. Retention was not significantly related to differences in response inhibition, working memory, global EF, peer preference, ADHD diagnostic status, or age. However, the retained sample at Wave 4 was higher in SES (*t* = 3.25, *p* =.004) and verbal IQ (*t* = 3.75, *p* =.001).

***Correlations.*** Table 1 reports descriptive statistics and correlations among key variables. Higher peer preference in childhood was associated with lower CPT commission error rates (i.e., higher inhibition) from adolescence to early adulthood (Waves 2–4; *r* = [-0.30, -0.23]) and lower ROCF/TCFT error proportion scores (i.e., higher global EF) from childhood to early adulthood (Waves 1–4; *r* = [-0.37, -0.32]). These correlations were of small to medium effects, increasing in magnitude across age. Regarding working memory, childhood peer preference was associated with higher backward digit span scores from childhood to emerging adulthood (Waves 1–3) but not in early adulthood (Wave 4). These latter correlations were slightly smaller than those of other EF measures and did not reveal a consistent trend across age (*r* = [0.15, 0.22]).

**Table 1:**
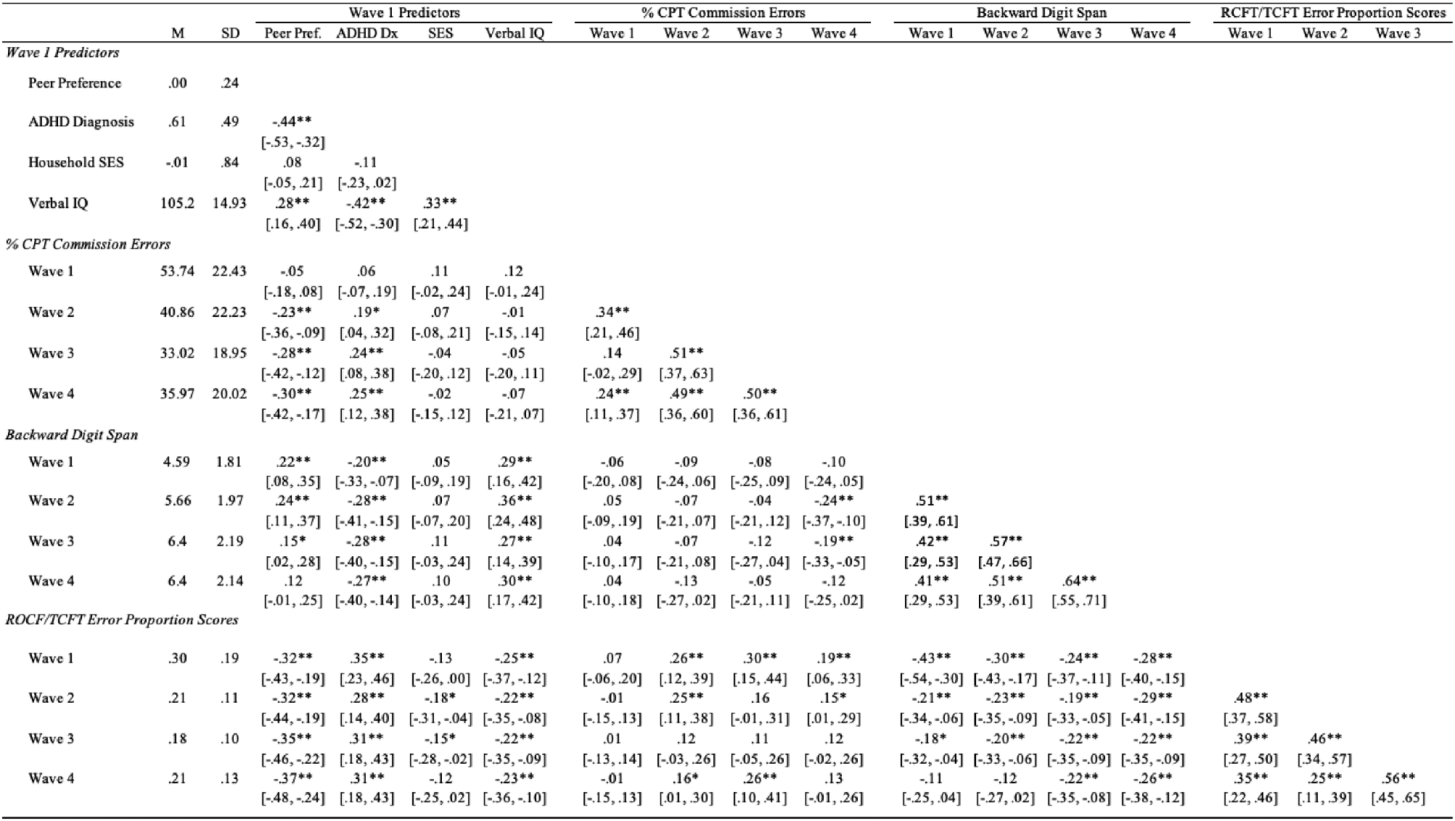
Means, standard deviations, and correlations with confidence intervals. *Note.* Point biserial correlations were used for associations with childhood ADHD diagnostic status (dichotomous); SES = socioeconomic status; CPT = Conners’ Continuous Performance Test; ROCF = Rey-Osterrieth Complex Figure; TCFT = Taylor Complex Figure Test; ^⁎^*p* < .05; ^⁎⁎^*p* < .01; ^⁎⁎⁎^*p* < .001

### EF Trajectories

***Unconditional Models.*** Comparing unconditional growth models, we found that a random intercept, random linear slope, fixed quadratic model best characterized the development of each EF measure from childhood to early adulthood, while still converging (*Δχ*^*2*^(1) = 37.45-108.29, *p* <.001). On average, EF measures significantly improved across development with decelerating growth over time, as indicated by the significant quadratic effect. This trend is consistent with past research indicating that EF development is steepest in childhood and adolescence with tapered growth extending into early adulthood (De Luca et al., 2003).

**Fig. 1:**
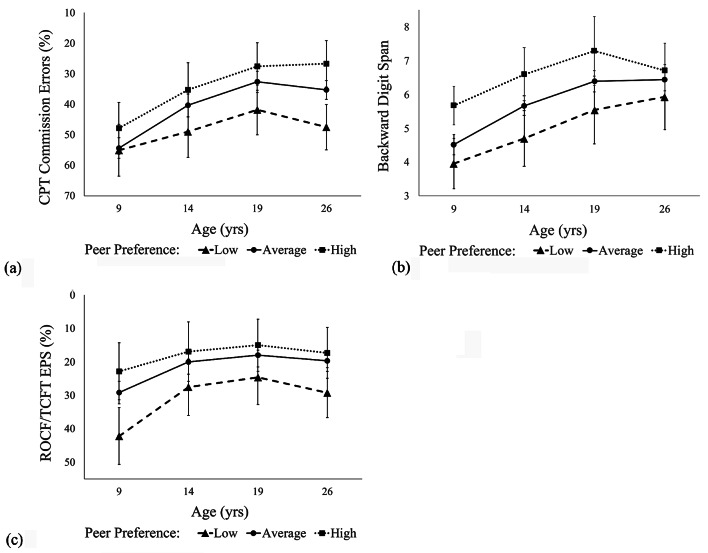
EF trajectories across development by childhood peer preference: (**a**) Response inhibition (**b**) Working memory (**c**) Global EF. *Note*. Data points reflect the average (**a**) response inhibition, (**b**) working memory, and (**c**) global EF skills of participants at each wave broken out by their level of peer preference in childhood (Low: < *M*-1*SD*; Average: within 1 *SD* of the mean; High: >*M*+1*SD*). The x-axis reflects the average age at each wave. Given that commission errors and error proportion scores are measures of EF deficits, their y-axes are flipped for consistency across measures. CPT = Conners’ Continuous Performance Test; ROCF = Rey-Osterrieth Complex Figure; TCFT = Taylor Complex Figure Test; EPS = error proportion score

***Response Inhibition.*** Including childhood peer preference and its interaction with age as predictors significantly improved model fit of response inhibition trajectories beyond covariates alone, *χ*^2^(2) = 13.36, *p* =.001 (Table 2). In particular, there was a significant interaction between childhood peer preference and linear age (*b* = -0.30, *SE* = 0.12, *p* =.01). Although girls did not show significant differences in baseline inhibition with respect to childhood peer preference, girls with higher childhood peer preference improved in response inhibition at a significantly faster rate than those with lower peer preference, as shown in Fig. 1a. Specifically, despite similar baseline levels at Wave 1, the CPT commission error rates of girls with high peer preference (i.e., one or more standard deviations above the mean) decreased to on average 26.70 (*SE* = 3.88) by Wave 4 (i.e., an average change of -21.18 between Waves 1 and 4; *M*_*age_W1*_ = 9.55 yrs; *M*_*age_W4*_ = 25.56 yrs), whereas the error rates of those with low peer preference (i.e., one or more standard deviations below the mean) only decreased to an average of 47.51 (*SE* = 3.80) by Wave 4 (i.e., an average change of only − 7.54 between Waves 1 and 4). Regarding covariates, there was a significant main effect of childhood ADHD diagnostic status: on average, girls with ADHD exhibited significantly higher commission error rates (i.e., poorer response inhibition) than comparisons (*b* = 6.78, *SE* = 3.17, *p* =.03). Neither childhood ADHD diagnosis nor other covariates (verbal IQ or SES) were related to differences in improvements in commission error rates over time. Note that interactions between predictors and quadratic slope were omitted from all models because they were too highly correlated with linear slope interaction terms (*r* >.90).

**Table 2:**
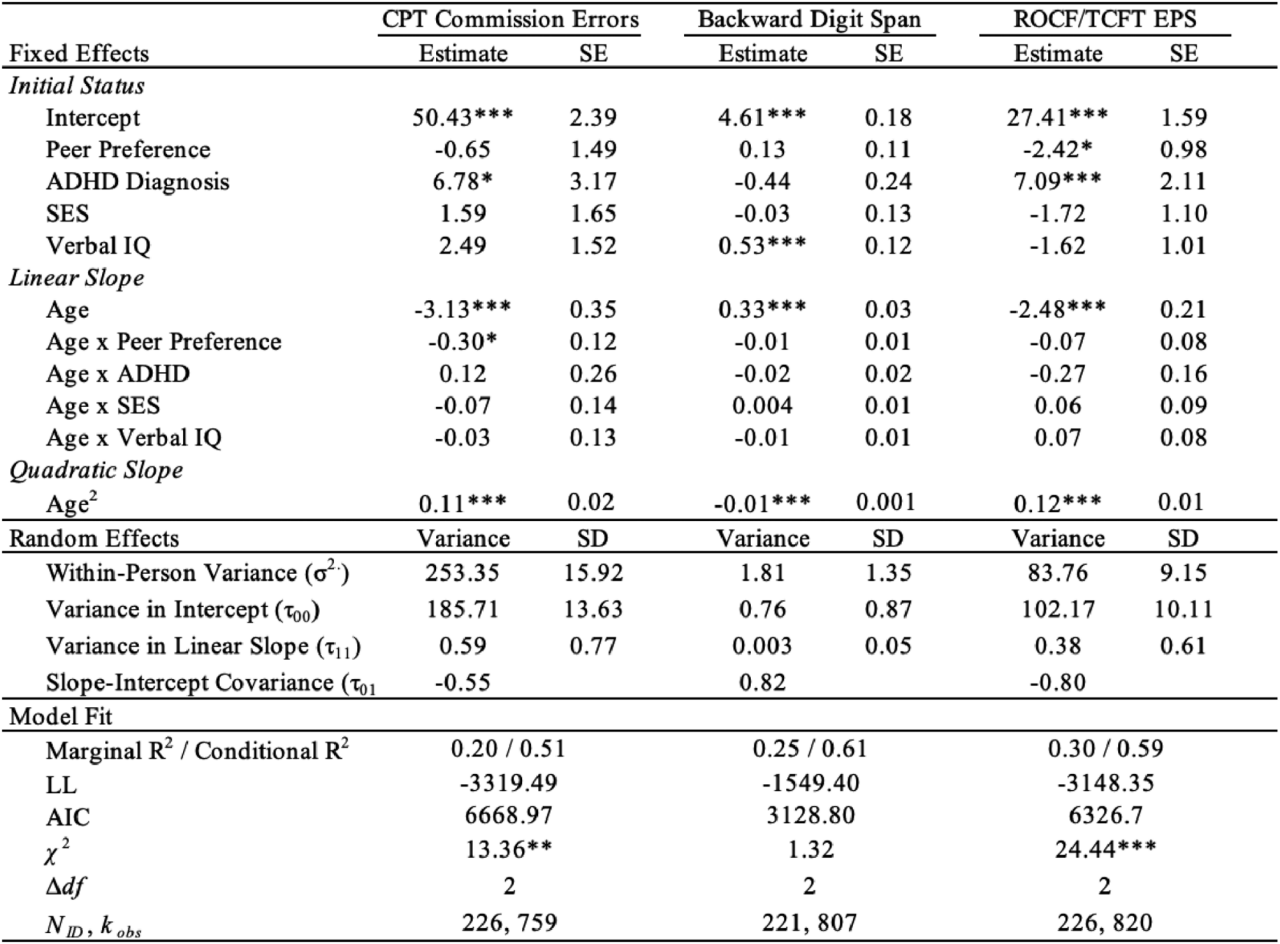
EF growth curves by peer preference. *Note.* Model fit comparisons are relative to equivalent models omitting peer preference and its interaction with age as predictors. Interactions between predictors and quadratic slope were omitted from all models due to high correlations with linear slope interaction terms (r > 0.90). CPT = Conners’ Continuous Performance Test; ROCF = Rey-Osterrieth Complex Figure; TCFT = Taylor Complex Figure Test; EPS = error proportion score; SES = socioeconomic status; LL = log likelihood ^⁎^*p* < .05; ^⁎⁎^*p* < .01; ^⁎⁎⁎^*p* < .001

***Working Memory.*** In contrast, adding childhood peer preference as a predictor did not significantly improve model fit beyond covariates for working memory, *χ*^2^(2) = 1.32, *p* >.05 (Table 2). Specifically, verbal IQ in childhood was related to significantly higher backward digit span scores across development (*b* = 0.53, *SE* = 0.12, *p* <.001). There were no significant interactions with age, suggesting that participants improved in working memory at similar rates across development with respect to peer preference and covariates (Fig. 1b).

***Global EF.*** As with inhibition, including childhood peer preference and its interaction with age as predictors significantly improved model fit, *χ*^2^(2) = 24.44, *p* <.001 (see Table 2). Specifically, childhood peer preference was related to significantly lower ROCF/TCFT error proportion scores (*b* = -2.48, *SE* = 0.21, *p* =.01), but this effect did not significantly vary across age (i.e., its interaction with linear age was non-significant). Additionally, childhood ADHD diagnostic status was related to higher error proportion scores (*b* = 7.09, *SE* = 2.11, *p* <.001) across development, also with no significant age interactions. Thus, both lower peer preference and ADHD diagnostic status were related to poorer global EF skills across development but were not predictive of differences in their rates of change over time, as shown in Fig. 1c.

***Peer Rejection vs. Acceptance.*** To explore the effects of childhood peer rejection vs. acceptance, we re-conducted analyses including these predictors in lieu of peer preference. Relations between childhood peer rejection and EF development mimicked those observed for peer preference. For response inhibition, there was a significant interaction between age and peer rejection in childhood, such that rejected girls exhibited less improvement in inhibitory control across development (*b* = 0.27, *SE* = 0.12, *p* <.05). Additionally, childhood peer rejection was related to lower global EF across development (*b* = 2.99, *SE* = 0.96, *p* <.01)—and this effect did not significantly vary across age. In contrast, peer acceptance in childhood was not related to response inhibition or global EF beyond its associations with childhood ADHD diagnostic status and peer rejection, respectively. Finally, neither peer rejection nor acceptance in childhood was related to working memory after adjusting for verbal IQ.

## Discussion

In this study, we examined prospective relations between childhood peer preference and the development of various EF skills—response inhibition, working memory, and global EF—among females with and without ADHD from childhood through their mid-to-late 20s. We hypothesized that higher peer preference in childhood would predict higher EF skills across development and significantly greater improvement in EF from childhood to early adulthood, adjusting for childhood ADHD diagnostic status, verbal IQ, and SES. Key findings, however, were domain specific: Higher childhood peer preference was (a) predictive of greater improvement in response inhibition across development, (b) not significantly related to working memory beyond its association with verbal IQ, and (c) related to higher global EF skills from childhood to early adulthood but not to differences in the rate of global EF development. Furthermore, relations between childhood peer preference and EF development were primarily driven by childhood peer rejection rather than peer acceptance. These findings help to address prior literature gaps by highlighting (a) the effects of both childhood peer preference and ADHD on EF development within a clinical sample enriched for ADHD, (b) the long-lasting impact of childhood peer preference on EF development extending into early adulthood, and (c) how this relation varies across distinct EF components, which we discuss further below.

To the best of our knowledge, this is the first study to evaluate the relation between childhood peer preference and EF development within a clinical sample enriched for EF deficits. Our findings are consistent with those of past studies using community samples, which highlight developmental links between peer relationships and EF in childhood (Burke et al., 2023; Holmes et al., 2016; Lecce et al., 2020). Additionally, we found that girls with ADHD exhibited poorer response inhibition and global EF across development, but unlike peer preference, ADHD was not predictive of differences in the rate of EF improvement from childhood to early adulthood. This finding suggests that childhood peer preference and ADHD are each related to EF development and perhaps in different ways. For example, although girls with ADHD exhibited poorer childhood response inhibition, as long as they are not rejected by their peers in childhood, they showed improvement in inhibition at similar rates to those of typically developing peers. In contrast, peer-rejected girls (regardless of ADHD-diagnostic status) showed significantly less improvement in inhibition over time, resulting in progressively worsening deficits in adolescence and early adulthood relative to non-rejected girls. This finding is consistent with Baumeister et al.’s theory (1998, 2005) that peer rejection threatens one’s fundamental need to belong, which may impair EF processes by depleting limited cognitive resources. This depletion is thought to result from the suppression of negative emotions induced by rejection and/or rumination over social problems—or how to compensate for poor relationships.

The finding that peer rejection, but not acceptance, was related to EF development is also consistent with longitudinal studies suggesting that deficits in self-regulation are more strongly related to negative than positive aspects of social experience (de Wilde et al., 2016; Ji et al., 2019). A key reason may be that peer rejection more actively threatens social belonging, resulting in heavier depletion of EF. However, considering that our sample is enriched for peer problems related to ADHD, further evaluation of the effects of distinct components of childhood peer relationships on EF development is warranted.

Our finding that childhood peer preference was significantly related to the development of response inhibition does not appear to be linked to differences in baseline levels of childhood inhibition, which were not significantly associated with concurrent peer preference. Indeed, experiences of rejection in childhood were predictive of less improvement in inhibition between childhood and early adulthood, at least within this naturalistic study. The influence of peer rejection on EF may be amplified across development because of increased sensitivity to perceived threats of belonging. For example, using fMRI data, Will et al. (2016) showed that chronic peer rejection in childhood is associated with increased neural responses to incidental exclusion in adolescence. In sum, peer rejection may not just be an outcome associated with ADHD but may also exacerbate inhibitory deficits related to the condition. We thus emphasize the importance of peer processes to adaptive EF development, which may serve as an important treatment target for this vulnerable population, as discussed further below.

Additionally, past research on the developmental links between peers and EF skills have largely focused on childhood (Burke et al., 2023; de Wilde et al., 2016; Lecce et al., 2020). Limited studies exploring effects on EF beyond childhood show a dwindling impact of peers on global EF in adolescence within community samples (e.g., Holmes et al., 2016)—or focus on the development of related skills, such as emotional regulation or inattention but not cold EF processes per se (Farley & Kim-Spoon, 2014; Ji et al., 2019; Stenseng et al., 2016). Here, we found that childhood peer rejection was related to poorer global EF and inhibition extending into early adulthood within a female sample enriched for ADHD, with rejected girls exhibiting less improvement in inhibition across development. Replication is needed to examine whether the persistence of EF deficits related to peer rejection generalizes beyond females with ADHD.

Finally, we found that peer effects on EF development vary across different EF components: childhood peer preference was predictive of differences in the rate of development only for response inhibition, but not working memory. Peers may be particularly important to the development of inhibitory control, which underlies not only the hyperactive-impulsive symptoms of ADHD (Miller et al., 2013) but other externalizing disorders as well (Beauchaine et al., 2017), potentially providing insight into the escalation of psychopathology among girls with ADHD.

In contrast, childhood peer preference was correlated with working memory, but this relation was no longer significant after adjusting for verbal IQ. It may be that past results linking peer preference and working memory are better explained by differences in verbal ability, considering that children with higher verbal skills tend to be more accepted by their peers (Troesch et al., 2016) and perform better on working memory tasks (Gathercole & Baddeley, 2014; Leonard et al., 2007). Yet it is also possible that this finding is an artifact of additional skills captured in our neuropsychological tests for each EF component, given that digit span tests assess *verbal* working memory, whereas continuous performance tasks and complex figure tests may emphasize visual-spatial and motor rather than verbal skills. Finally, our measures of working memory and verbal IQ were both from the WISC, which may have yielded shared method variance. Further replication is needed to examine the effect of peer relationships on the development of different EF components, using multiple tasks to assess each EF domain.

### Limitations and Implications

Several study limitations are noteworthy. First, regarding EF, this construct is inherently complex, involving multiple interconnected components that show increasing differentiation across development (Best & Miller, 2010; Wiebe et al., 2008). We focused on the development of response inhibition, working memory, and global EF separately, but future studies may consider other EF components as well, such as set shifting or cognitive flexibility, and their interrelations across development. Even more, evaluation of EF processing in heightened motivational or emotional contexts (i.e., hot EF) is particularly warranted considering past research that peer interactions may differentially affect hot vs. cold EF skills (King et al., 2018). As well, although informant reports of EF skills may be skewed by biases related to child social preference, using laboratory-based neuropsychological measures of EF may constrain ecological validity (Burgess et al., 2006), especially considering our use of single task measures for each EF component. Using aggregate measures across multiple neuropsychological tasks for each EF domain may enhance reliability and generalizability (Perugini et al., 2000).

Second, as for our core predictor of peer preference, we assessed this using gold-standard peer sociometric interviews, but these were held during summer programs involving an atypically high ratio of ADHD to comparison girls. Although ADHD and comparison girl nominations were similar overall, girls with ADHD rated others with ADHD slightly less negatively than did comparison girls (Blachman & Hinshaw, 2002)*.* We therefore expect that the present results may actually underestimate the level of peer rejection experienced by girls with ADHD: Findings in more representative samples may be stronger than those herein. Additionally, a minority of girls with ADHD used stimulant medication during part of the summer camp, which may have affected sociometric evaluations. It is also possible that peer ratings may have been impacted by participant interactions outside the summer camps, although participants were selected and put into different summer camp groups to minimize any potential impact of prior reputation. Furthermore, we exclusively relied on peer nominations in childhood, even though peer relationships continue to evolve across development and later relationships may help correct or exacerbate childhood EF deficits. Future studies should consider assessing repeated measures of both EF and peer preference across development to help inform the timing of social interventions and further address the directionality of these relations. We note, however, that sociometric assessments are logistically challenging beyond grade school.

Furthermore, although we focused on the influence of peers, clearly other social relationships also provide important contexts for EF development. For example, several past studies highlight the impact of early parent-child relationships (Fay-Stammbach et al., 2014; Valcan et al., 2018), with recent research also emphasizing the importance of teacher-child relationships (de Wilde et al., 2016). Finally, although our finding that childhood peer preference predicted differences in inhibitory growth across development is compelling, randomized control trials examining the impact of peer interventions on EF development are needed to test the causality of this relation, identify key underlying mechanisms, and refine potential interventions. Further exploration of this relation within mixed-gender, clinical samples is also needed to assess the generalizability of our findings beyond girls with ADHD.

Despite these limitations, to the best of our knowledge the present investigation is the first to evaluate the relation between childhood peer preference and the development of various EF components (a) extending from childhood to early adulthood or (b) within a longitudinal ADHD-enriched sample. Our results highlight the long-lasting effects of both childhood peer preference and ADHD on EF development and how these differ across distinct EF components. For example, childhood peer rejection may be particularly important to the development of response inhibition, with rejected girls showing significantly less improvement in inhibition from childhood to early adulthood. These findings suggest that school-based interventions aimed at reducing peer rejection and promoting inclusive classroom environments, such as the Making Socially Accepting Inclusive Classrooms program (Mikami et al., 2013), may be ideal candidates for curtailing the escalation of inhibitory deficits associated with ADHD, as well as related externalizing and self-harmful behaviors (Beauchaine et al., 2019). Further research examining such interventions may also help address limitations of EF training programs, such as the lack of transfer effect (Diamond & Ling, 2016) along with treatment access barriers associated with interventions focused on the family or individual (Kazdin, 2011; Saxena et al., 2007).

## Data Availability

These data emanate from a large, multi-wave and multi-measure longitudinal study. Thus, it is not possible to post all these data online. However, we will make the data used in this manuscript available upon request.
